# Supplementary material for: A potent and selective inhibitor for the modulation of MAGL activity in the neurovasculature
Source: PLoS One. 2022 Sep 9;17(9):e0268590. doi: 10.1371/journal.pone.0268590 (PMC9462760; doi:10.1371/journal.pone.0268590)
Supplement: S1 Table — *Values in parentheses are for highest-resolution shell. (DOCX) [file pone.0268590.s001.docx]

**Supplementary Table 1.** Data collection and refinement statistics for human MAGL compound 432 complex

|  | human MAGL compound 432 complex |  |
| --- | --- | --- |
| **Data collection** |  |  |
| Space group | C222_1_ |  |
| Cell dimensions |  |  |
| *a*, *b*, *c* (Å) | 89.96, 127.45, 63.03 |  |
| α, β, γ (°) | 90, 90, 90 |  |
| Resolution (Å) | 1.16 (1.26-1.16) |  |
| *R*_sym_ | 0.057 (0.80) |  |
| *I* / σ*I* | 11.27 (1.09) |  |
| CC(1/2) | 0.999 (0.584) |  |
| Completeness  Redundancy | 99.8 (99.5)  6.39 (5.99) |  |
|  |  |  |
| **Refinement** |  |  |
| Resolution (Å) | 63.72 – 1.16 |  |
| No. reflections | 122098 |  |
| *R*_work_ / *R*_free_ | 17.72/18.71 |  |
| No. atoms |  |  |
| Protein | 2306 |  |
| Water  Ligand | 377  28 |  |
| *B*-factors |  |  |
| Protein | 18.12 |  |
| Water  Ligand | 34.30  19.25 |  |
| R.m.s. deviations |  |  |
| Bond lengths (Å)  Bond angles (°) | 0.008  0.950 |  |
|  |  |  |
|  |  |  |

*Values in parentheses are for highest-resolution shell.
