# Supplementary material for: A potent and selective inhibitor for the modulation of MAGL activity in the neurovasculature
Source: PLoS One. 2022 Sep 9;17(9):e0268590. doi: 10.1371/journal.pone.0268590 (PMC9462760; doi:10.1371/journal.pone.0268590)
Supplement: S2 Table — Mm = Mus musculus, Hs = Homo sapiens. (DOCX) [file pone.0268590.s002.docx]

**Supplementary Table 2.** Droplet digital qRT-PCR TaqMan assays. Mm = *Mus musculus*, Hs = *Homo sapiens*.

| Target (species) | Catalogue ID | Fluorophore |
| --- | --- | --- |
| *Mgll* (Ms) | Mm00449274_m1 | FAM |
| *IL-1β* (Ms) | Mm00434228_m1 | FAM |
| *IL-6* (Ms) | Mm00446190_m1 | FAM |
| *LCN2* (Ms) | Mm01324470_m1 | FAM |
| *TNF* (Ms) | Mm00443258_m1 | FAM |
| *EMC7* (Ms) | Mm00505280_m1 | VIC |
|  |  |  |
| *Mgll* (Hs) | Hs00996004_m1 | FAM |
| *EMC7* (Hs) | Hs00220077_m1 | VIC |
