# Supplementary figures and images for: A potent and selective inhibitor for the modulation of MAGL activity in the neurovasculature
Source: PLoS One. 2022 Sep 9;17(9):e0268590. doi: 10.1371/journal.pone.0268590 (PMC9462760; doi:10.1371/journal.pone.0268590)

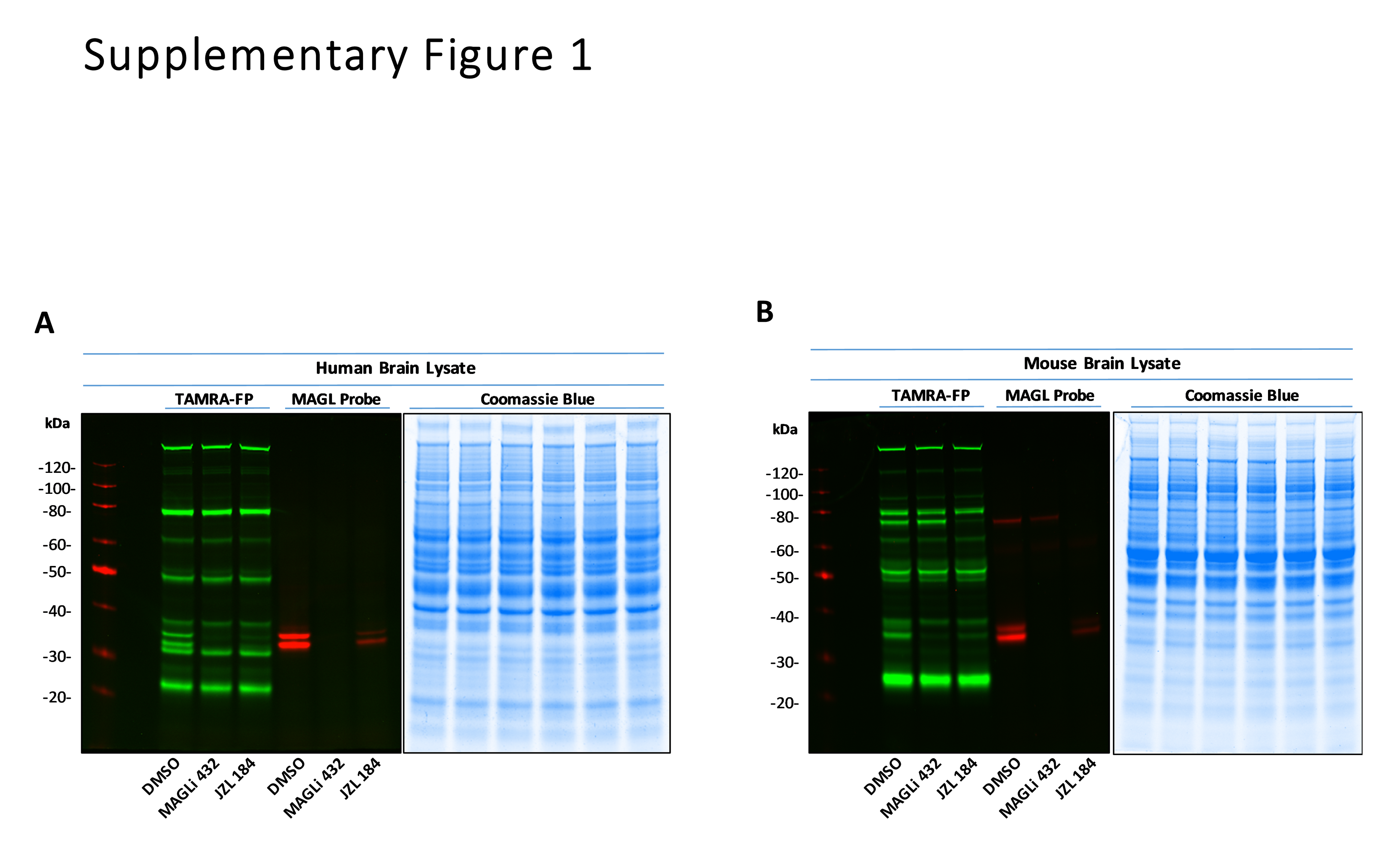

Supplement: S1 Fig — Selectivity of MAGLi 432 in human (A) and mouse (B) brain lysates was determined by gel based, competitive Activity Based Protein Profiling (ABPP). Brain lysates were incubated with either DMSO, 10 μM MAGLi 432 or 10 μM JZL 184 for 30 mins and then incubated with either broad serine hydrolase activity based probe, TAMRA-FP (green) or MAGL-specific probe (red) before samples were loaded on an SDS-PAGE gel and proteins separated by electrophoresis. Gels referenced from Fig 1 were counterstained with Coomassie Brilliant Blue to visualize total protein per lane (n = 2). Normalized MAGL activity quantified as the average signal intensity for each probe divided by total protein bands observed at the corresponding MW to MAGL bands (Coomassie Blue signal) (Fig 1). (TIF) [file pone.0268590.s003.tif]

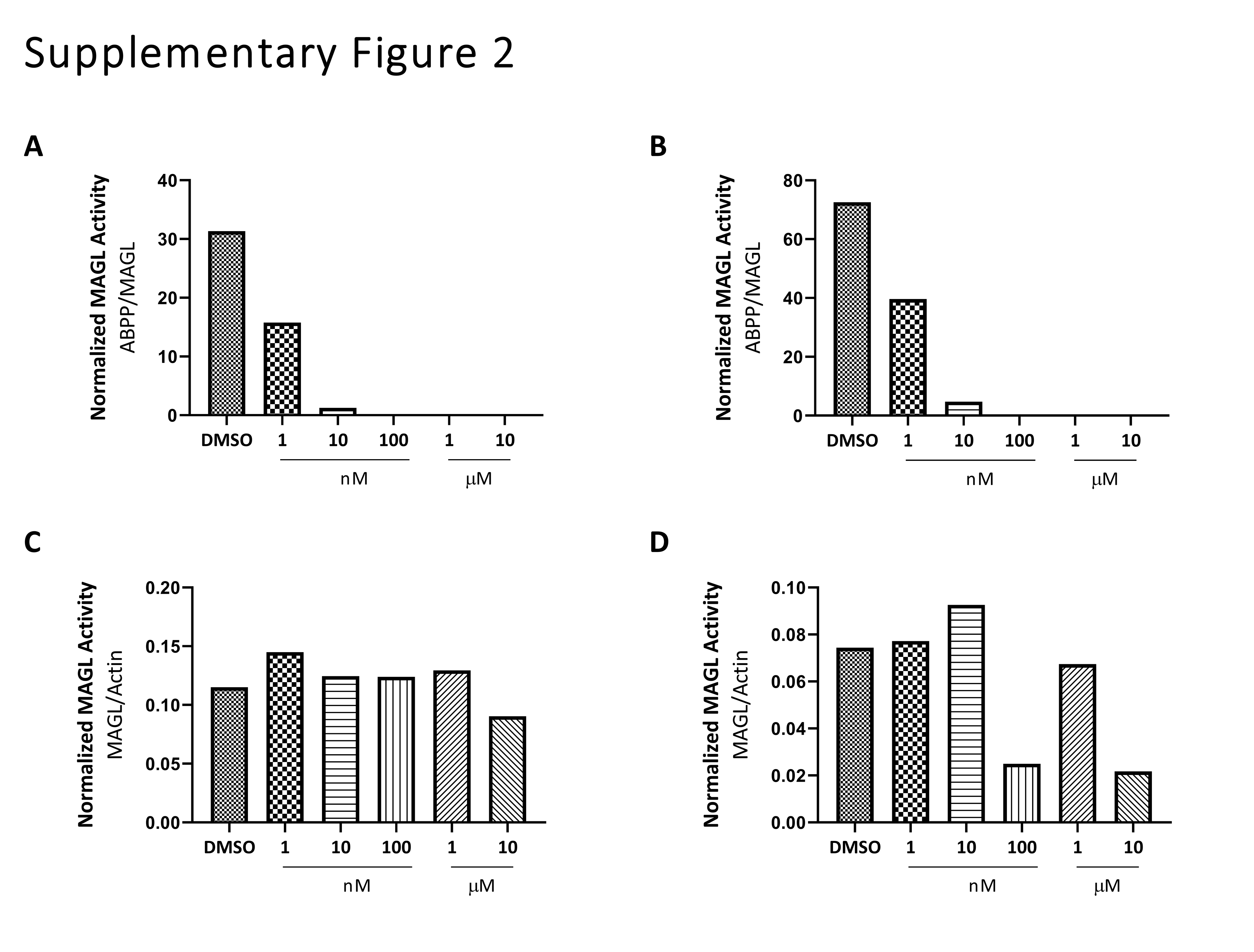

Supplement: S2 Fig — Assessment of MAGLi 432 potency was measured by incubation of ascending doses of MAGLi 432 in human brain lysates (A, C) and mouse brain lysates (B,D) as measured by competitive ABPP with the MAGL-specific probe. Average signal intensity of active MAGL and total MAGL protein in lysates quantified (A, B) as total detectable active MAGL band signal (ABPP) over total MAGL band signal (WB) or (C, D) total MAGL protein over total β-actin band signal (n = 2). (TIF) [file pone.0268590.s004.tif]

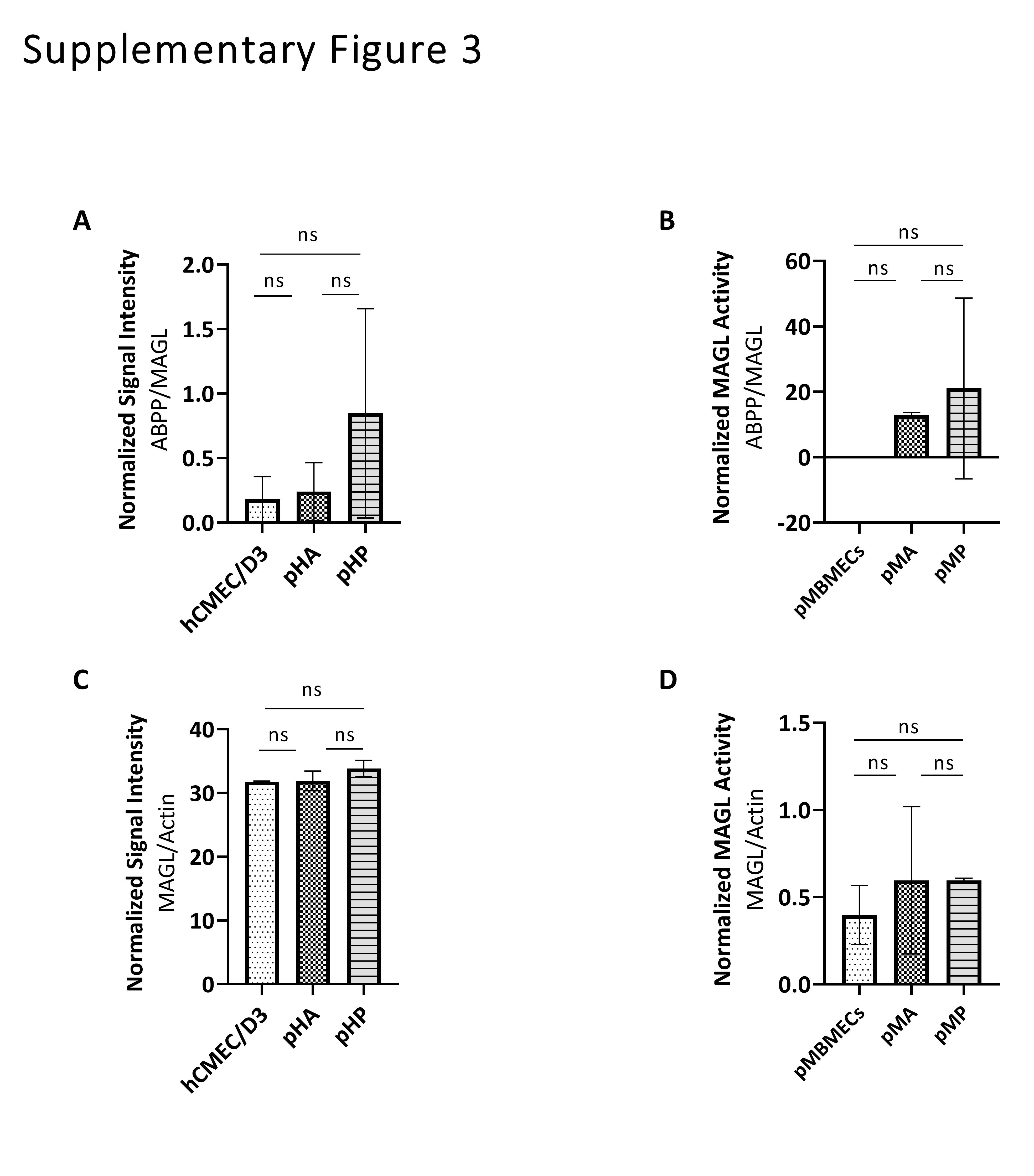

Supplement: S3 Fig — Active MAGL was measured by incubation with the MAGL-specific probe via ABPP. Total MAGL protein expression and β-actin were determined by WB. Average signal intensity of active MAGL and total protein in lysates quantified (A, B) as total detectable active MAGL band signal (ABPP) over total MAGL band signal (WB) or (C, D) total MAGL protein over total β-actin band signal (n = 2). Results are reported as mean ± SD, one-way ANOVA (ns = not significant, * = p < 0.05, ** = p < 0.01, *** = p < 0.001, **** = p < 0.0001). (TIF) [file pone.0268590.s005.tif]

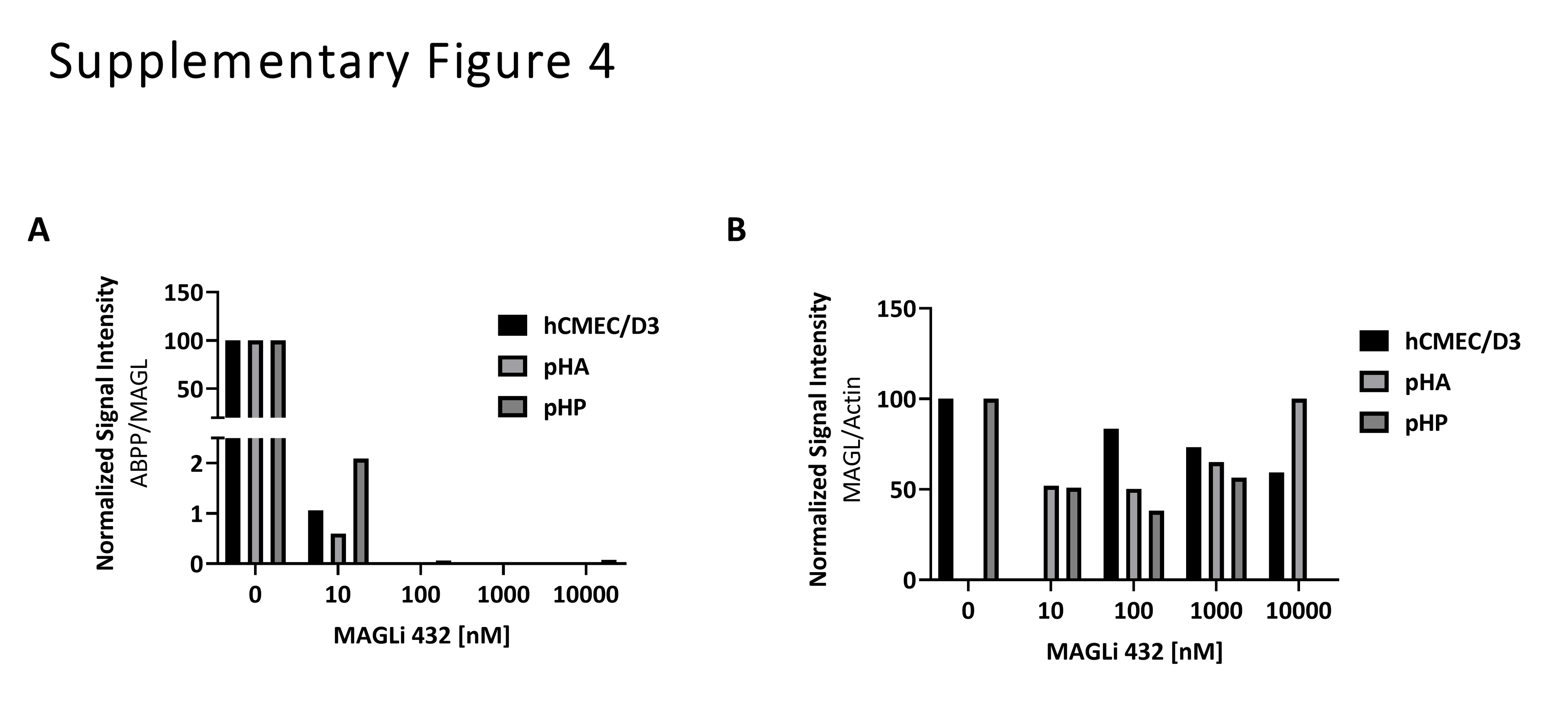

Supplement: S4 Fig — Assessment of MAGLi 432 potency in vitro was measured by incubation of ascending doses of MAGLi 432 (10nM, 100nM, 1μM, 10μM) in human NVU Cells for 6 hours. Cell lysates from each group were then collected and then incubated with the MAGL-specific probe. Proteins were then separated by gel electrophoresis and in gel fluorescence was measured. Average signal intensity of active MAGL and total MAGL protein in lysates quantified (A) as total detectable active MAGL band signal (ABPP) over total MAGL band signal (WB) or (B) total MAGL protein over total β-actin band signal, with the highest signal normalized to 100%. (n = 2). (TIF) [file pone.0268590.s006.tif]

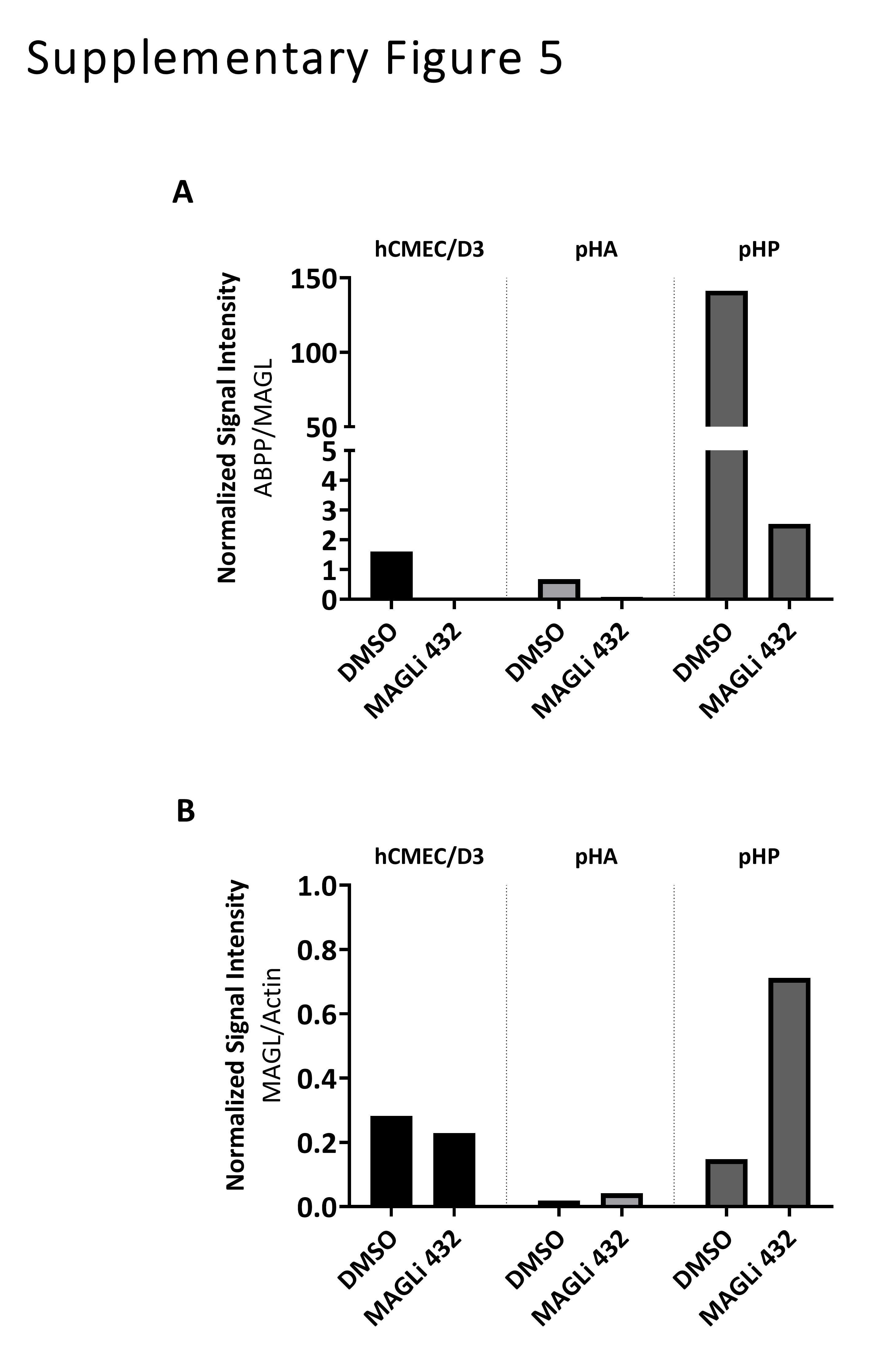

Supplement: S5 Fig — Assessment of MAGLi 432 in vitro was measured by incubation of DMSO (solvent control) or 1μM MAGLi 432 with human NVU cell cultures for 6 hours. Cell lysates from each group were then collected and then incubated with the MAGL-specific probe. Proteins were then separated by gel electrophoresis and in gel fluorescence was measured. Average signal intensity of active MAGL and total MAGL protein in lysates quantified (A) as total detectable active MAGL band signal (ABPP) over total MAGL band signal (WB) or (B) total MAGL protein over total β-actin band signal. (n = 2). (TIF) [file pone.0268590.s007.tif]

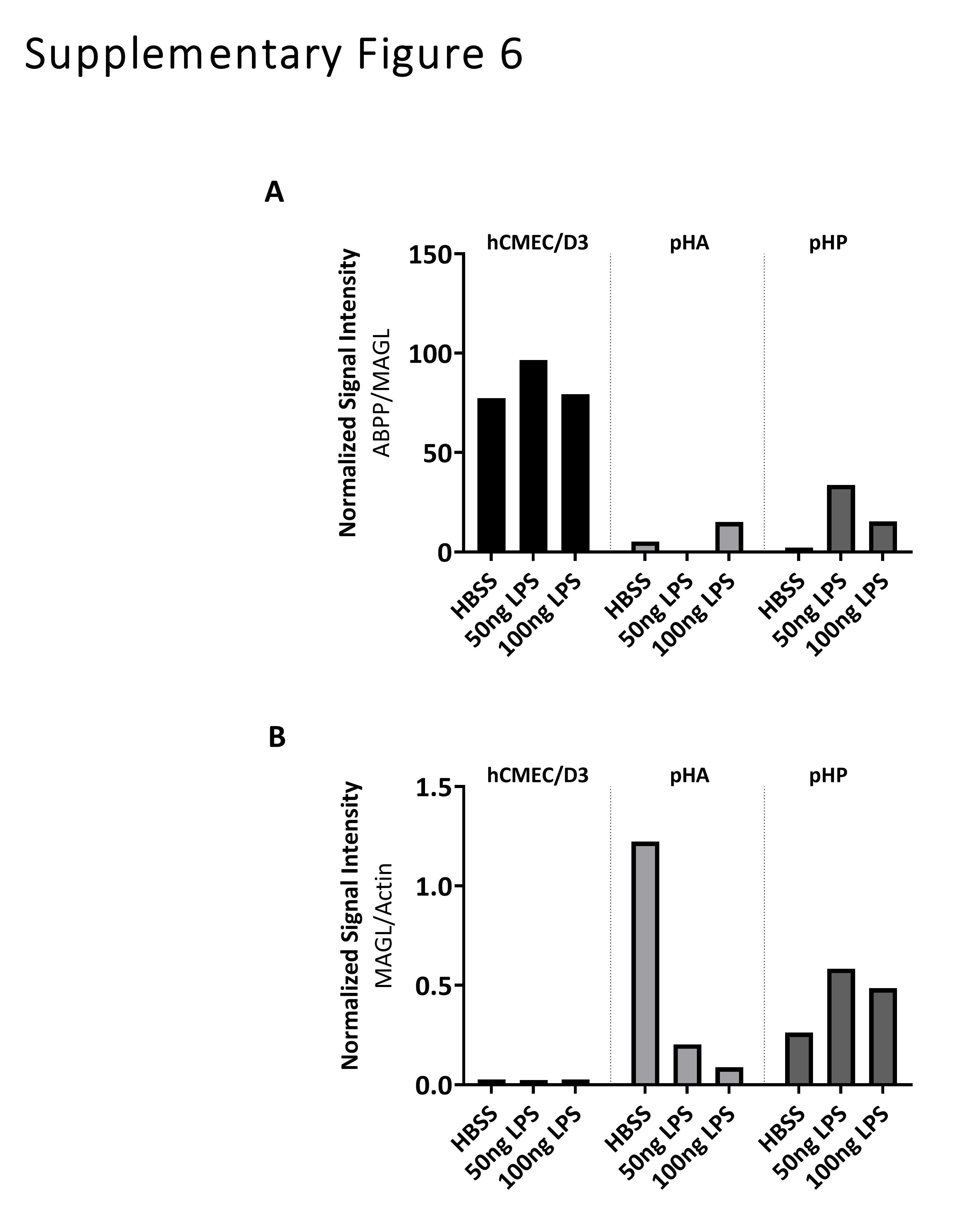

Supplement: S6 Fig — Quantification of effects of HBSS (solvent control) and LPS (50ng and 100ng) in vitro by MAGLi 432 in human NVU cells (hCMEC/D3, pHA and pHP) (from Fig 4A and 4B). Assessment of MAGLi 432 potency in vitro was measured by incubation of 1μM MAGLi 432 in human NVU Cells for 6 hours. Cell lysates from each group were then collected and then incubated with the MAGL-specific probe. Proteins were then separated by gel electrophoresis and in gel fluorescence was measured. Average signal intensity of active MAGL and total MAGL protein in lysates quantified (A) as total detectable active MAGL band signal (ABPP) over total MAGL band signal (WB) or (B) total MAGL protein over total β-actin band signal. (n = 2). (TIF) [file pone.0268590.s008.tif]

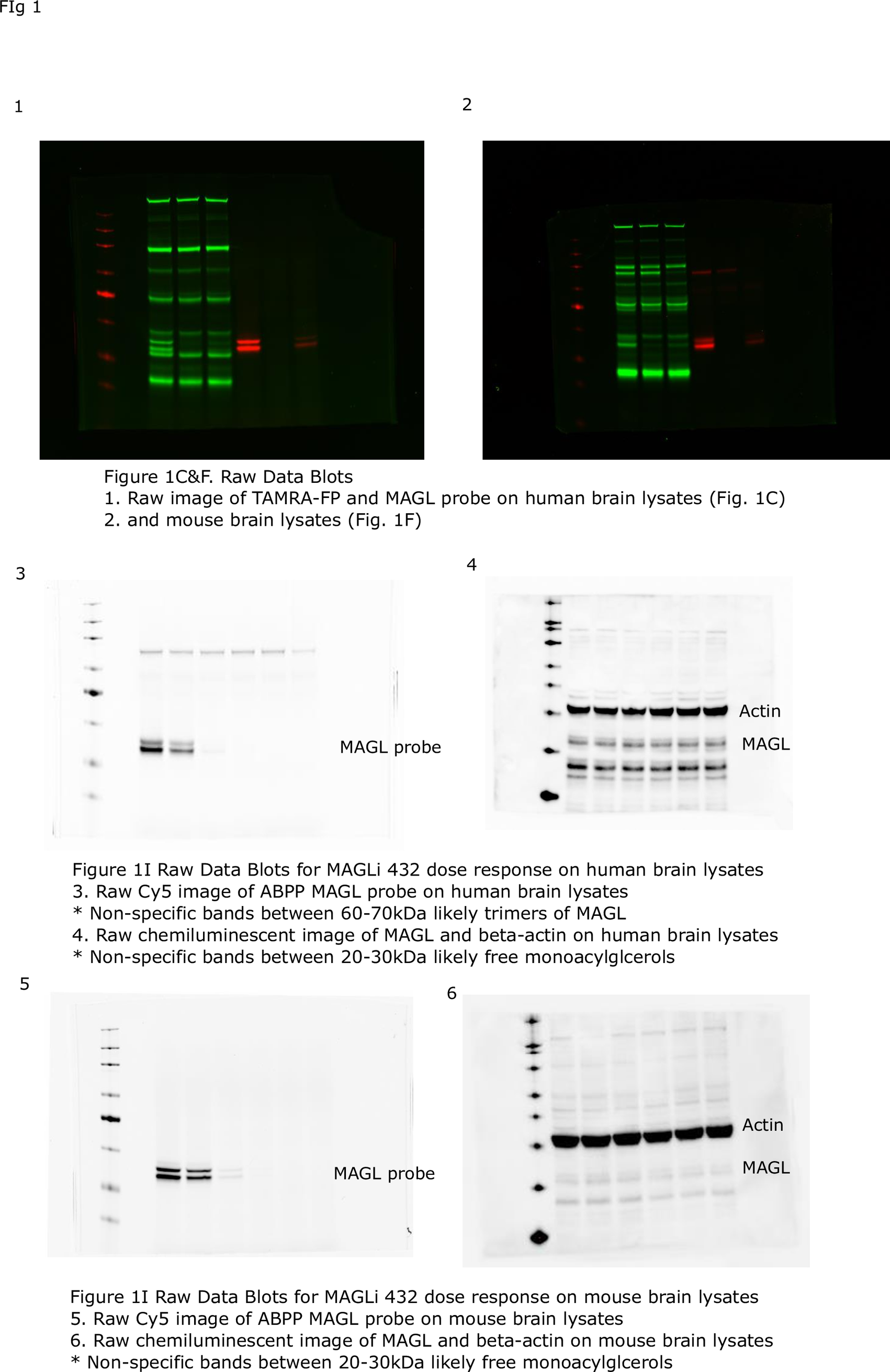

Supplement: S1 Raw images — (TIF) [file pone.0268590.s009.tif]
